# Effects of a short-term temperature increase on arthropod communities associated with pastures

**DOI:** 10.3897/BDJ.11.e107385

**Published:** 2023-10-05

**Authors:** Sophie Wallon, Noelline Tsafack, Gabor Pozsgai, Catarina Melo, Paulo A. V. Borges, Rui Elias

**Affiliations:** 1 cE3c- Centre for Ecology, Evolution and Environmental Changes/Azorean Biodiversity Group, CHANGE – Global Change and Sustainability Institute, School of Agricultural and Environmental Sciences, University of the Azores, Rua Capitão João d´Ávila, Pico da Urze, 9700-042, Angra do Heroísmo, Azores, Portugal cE3c- Centre for Ecology, Evolution and Environmental Changes/Azorean Biodiversity Group, CHANGE – Global Change and Sustainability Institute, School of Agricultural and Environmental Sciences, University of the Azores, Rua Capitão João d´Ávila, Pico da Urze, 9700-042 Angra do Heroísmo, Azores Portugal; 2 Regional Secretariat of Environment and Climate Change, Project LIFE BEETLES (LIFE 18NAT/PT/000864), Rua do Galo n118, 9700-040, Angra do Heroísmo, Azores, Portugal Regional Secretariat of Environment and Climate Change, Project LIFE BEETLES (LIFE 18NAT/PT/000864), Rua do Galo n118, 9700-040 Angra do Heroísmo, Azores Portugal; 3 CFE – Centre for Functional Ecology, 3001-401 Coimbra, Portugal CFE – Centre for Functional Ecology 3001-401 Coimbra Portugal; 4 IUCN SSC Mid-Atlantic Islands Invertebrate Specialist Group, Angra do Heroísmo, Azores, Portugal IUCN SSC Mid-Atlantic Islands Invertebrate Specialist Group Angra do Heroísmo, Azores Portugal

**Keywords:** invertebrates, Azores, climate change, grasses, islands, species diversity

## Abstract

The impact of climate change on islands is expected to cause dramatic consequences on native biodiversity. However, limited data are available for arthropod communities in island agroecosystems. In this study, we simulate a small-scale climatic change (average of +1.2°C), using Open Top Chambers (OTCs) in forage crops in the Azores Archipelago (Portugal) and test the responses of arthropod communities associated with intensively-managed pastures. At three sites, twenty 1 x 1 m plots were established: 10 treatment plots with OTCs and 10 control plots. Arthropods were sampled with pitfall traps on two sampling events (winter and summer of 2020). When considering all species collected, arthropods' abundance was lower in OTCs. Specific taxa, namely spiders and beetles, showed a fast response to the OTCs' presence. The assemblage of non-indigenous spiders well adapted to pastures showed a significant difference in diversity with a slightly greater richness, but lower abundance inside the warmer plots. However, the presence of OTCs resulted in a decrease in beetle richness and abundance. This decline may be attributed to the multiple effects of warming. Therefore, it is imperative to conduct further investigations to elucidate the ecological processes that underlie the observed patterns.

## Introduction

Climate change is happening at a fast pace and the results are the increase in temperature (Arnell et al. 2019, Portner et al. 2022), change in precipitation patterns (Ohba and Sugimoto 2018, Papalexiou and Montanari 2019) and the increase in the frequency and severity of extreme natural events (Banerjee et al. 2018, Hettiarachchi et al. 2018, Yin et al. 2018, Myhre et al. 2019, Papalexiou and Montanari 2019). Those changes are altering ecosystems and affecting biodiversity (Sharma and Dhillon 2018, Habibullah et al. 2021). Agroecosystems which support food production are no exceptions and they also can be impacted in several ways: climate change can cause heat stress on crops and reduce yields, altered precipitation regimes jeopardise available water in the critical growth periods and natural disasters can cause direct damage (Schoene and Bernier 2012, Cook et al. 2018, Craig et al. 2019, Elahi et al. 2022).

In particular, island environments are especially sensitive to climate change and have been identified amongst the most vulnerable ecosystems to climate shifts and extremes (Nurse et al. 2014). On islands, the vulnerability is defined by the system’s capacity to respond to modifications in the abiotic environment and their adaptation to climate change can be challenging (Santos et al. 2004, Harter et al. 2015, Taylor and Kumar 2016, Vogiatzakis et al. 2016). For instance, in the Azorean Islands (nine islands located in the North Atlantic), the main consequence of climate change is the increase in temperature and the decrease in rain (Brito de Azevedo and Pereira 1998, Brito de Azevedo et al. 1999). The two respective scenarios, RCP 4.5 and RCP 8.5 (PRAC 2017), predict for the Azores an increase in temperature between 0.78°C and 0.90°C until 2039 and a further increase by between 1.5°C and 2.8°C until the end of the century. Those changes in climate may also result in shifts in cloud layer altitudes, which, in turn, may alter altitudinal zonation of the vegetation and different species assemblages, mostly promoting species losses (Harter et al. 2015). According to projection models, the distribution of the unique biota of the Azores will be affected (Ferreira et al. 2016, Patiño et al. 2016) by the loss of suitable climatic space due to climate change and should be taken into account for the design of protected areas (Ferreira et al. 2019). Following the “Programa Regional para as Alterações Climáticas” (Regional Programme for Climate Change) (PRAC 2017), the most important impacts associated with climatic factors in the Azores are the stress induced on ecosystems and natural resources which causes the reduction of agriculture production in situations of drought, the reduction of quality of the pasture and fodder and the expansion of some insect pests.

In the Azores Archipelago, around 56% of the land is dedicated to agroecosystems, of which 46% are permanent pastures (Massot 2015), representing 88% of usable agricultural area (Government of the Azores 2023). The grasslands and pastures cover about 41.5% of Terceira (Cruz et al. 2007, Reis and Ponce Dentinho 2015), the third largest island in the Archipelago. They support semi-extensive dairy and beef cattle farming, both by fodder production and direct grazing (Massot 2015). Although the impacts of global change on grasses in pastures are already widely studied and relationships between nutritive parameters and environmental factors that can alter grass quality and its digestibility for cattle are confirmed (Berauer et al. 2020, Hart et al. 2022, Melo et al. 2022), there are still limited data available related to the impact of climate change on Azorean agroecosystems. However, it is likely that these, like native ecosystems, will also become increasingly threatened by climate change. Since arthropods provide a wide range of ecosystem services (e.g. pollination, biological control) and also cause ecosystem disservices (e.g. crop damage, pest outbreaks), how their diversity is changing with the changing climate should be of utmost importance in agroecosystems (Zhang et al. 2007, Allan et al. 2015, Borges et al. 2021, Ferrante et al. 2023).

In the last years, a plethora of studies have shown the impact of global change on arthropods in grasslands, pinpointing how climate change can affect arthropod communities and alter their diversity and/or composition (Gobbi et al. 2006, Buchholz et al. 2012, Barnett and Facey 2016, Pitta et al. 2019). Fully controlled, short-term experiments (less than two years) already indicate the impact of increased temperature on arthropod abundances and richness (Molina-Montenegro et al. 2008, Hågvar and Klanderud 2009, Buchholz et al. 2012, Sohlström et al. 2022). Despite the fact that experiments have already highlighted changes occurring in arthropod communities in different grassland and pasture in the world under climate change and that the arthropod communities of Azorean pastures have been investigated in detail (Borges and Brown 1999, Borges and Brown 2004, Borges 2008, Cardoso et al. 2009, Rigal et al. 2017), no study has yet scrutinised the impact of climate change (e.g. increase in temperature) on those communities in the Azores. In this short-term in situ experiment, we aim to test the impacts of increasing temperature on arthropod communities in pastures on Terceira, the third largest island of the Azores Archipelago, using Open Top Chambers (OTCs).

We hypothesise that warming will affect arthropod communities and we aim to identify which groups or species of arthropods are more likely to respond positively or negatively to an increase in temperature. We address the following research questions:

i) Does the increase in temperature within OTCs change the species composition? Due to the small-scale and the short-term of the experiment, we predict little or no changes in the species composition and, consequently, that communities will remain highly similar between the two treatments.

ii) Does the increase in temperature in OTCs change the total abundance of arthropods? Based on previous studies, we expect higher total arthropod abundance in OTCs than in the control plots with ambient temperature.

iii) Does the increase in temperature impact the relative abundances of species? We expect shifts in the ratio of common, rare and dominant species, especially an increasing dominance of a few, thermophilus, species.

## Material and methods

### Study area

**Figure 1.**
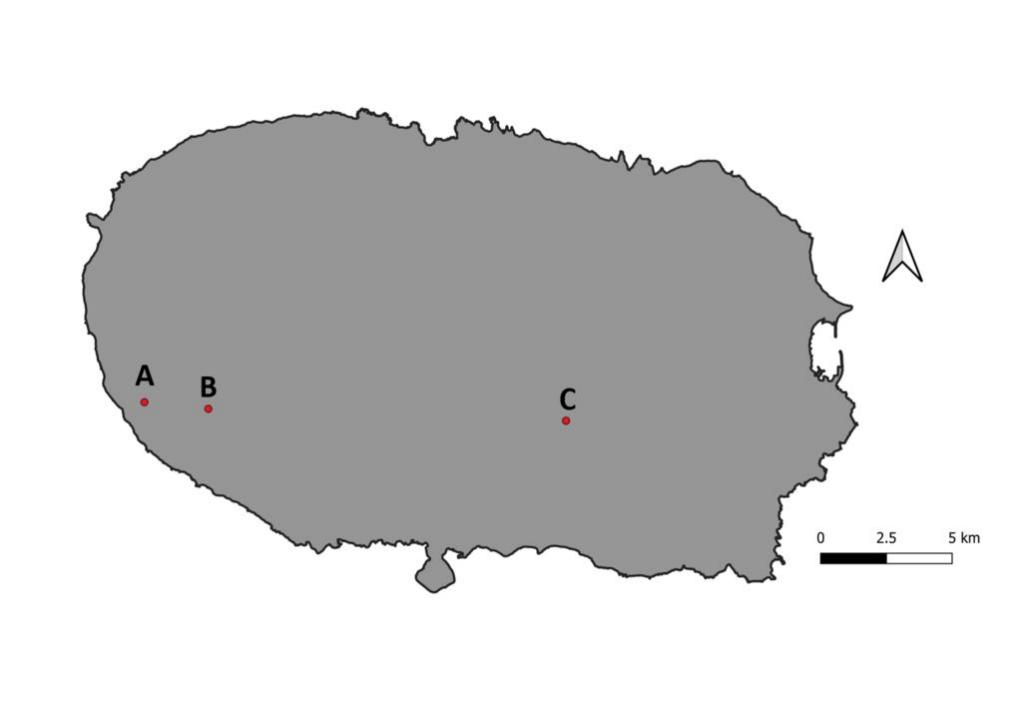
Locations of each field on the Island of Terceira. Fields A, B and C are located respectively at 186 m, 301 m and 386 m a.s.l.

The study was conducted in three intensively-managed experimental pasture fields on Terceira Island (area 402 km^2^ and maximum elevation of 1023 m), located in the Azores Archipelago in Portugal (38°37’ N–38°48’ N, 27°02’ W–27°23’ W). The three fields (marked as A, B and C) were located at three different altitudes: 186 m a.s.l. (latitude: 38.703596°N; longitude: -27.353805°W), 301 m a.s.l. (latitude: 38.701639°N; longitude: - 27.325783°W) and 386 m a.s.l. (latitude: 38.697770°N; longitude: -27.170075°W), respectively (Fig. 1). All fields are considered as intensive pasture, dominated by the Italian ryegrass, *Loliummultiflorum* Lam. (Poaceae) in fields A and B and dominated by the common velvet grass, *Holcuslanatus* L. (Poaceae) in field C.

### Experimental design

An in situ experiment was set up using Open Top Chambers (OTCs). OTCs are widely used on climate change investigation in order to modify abiotic conditions in situ and simulate an increase in temperature (Aronson and McNulty 2009). The OTC panels act as a wind shield, minimising the amount of heat lost through convection and the open top allows rainfall as well as air circulation, creating small eddies (Hollister et al. 2022).

**Figure 2.**
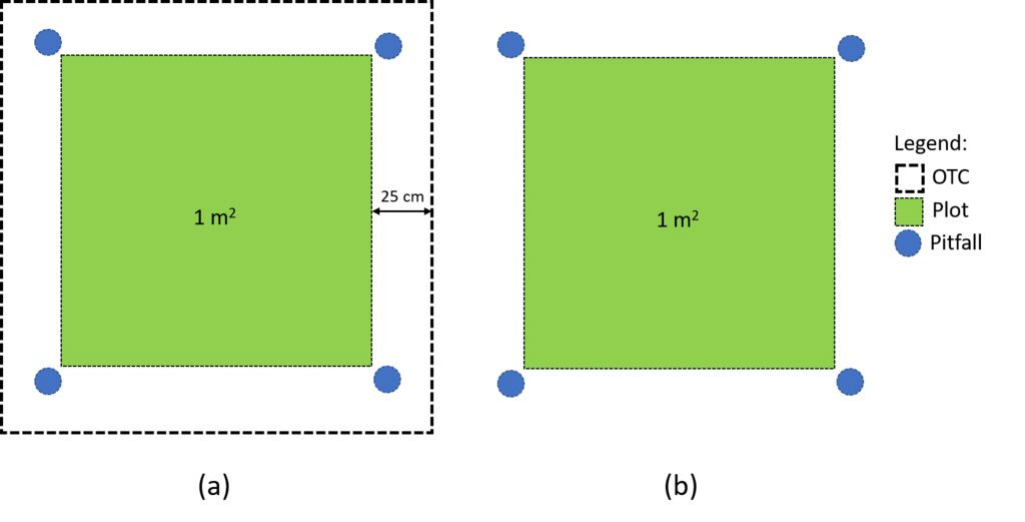
Setup of an OTC plot (a) and a control plot (b).

In each field, twenty (1 x 1 m) plots were set up on a grid pattern with 1.5 m space between each plot (Fig. 2). Of the twenty plots, ten were randomly chosen as control and the other ten were surrounded by OTCs. Data loggers (Easy Log: EL-USB-2) were set up to collect the temperature data inside the OTCs, as well as in the control plots. The temperature inside the OTCs was on average 1.2ºC higher than in the control plots. OTCs were set up in order to include the 1 x 1 m plot as well as a margin of 25 cm all around the plot. This allowed collectors to access the plot without damaging it, as well as to set up the pitfall traps on each outside corner of the plot, but still inside the OTCs. The OTCs were also slightly raised from the ground (around 5 cm) in order to allow crawling arthropods to disperse freely around the whole sampling area.

The sampling was carried out during two seasons (winter and summer of 2020), before the grass had been mown. No cattle were allowed inside the sampling area. It is important to note that the experimental set-up (control and OTCs) was mounted the whole year round. Thus, the OTCs and control plots were not moved between the two sampling seasons.

### Arthropod sampling and identification

For this study, we focused on arthropod communities associated with intensive pasture management. As OTCs represent a physical barrier for flying insects and can induce bias into the results, the present study focuses on crawling arthropods. As pitfall traps target crawling arthropods, they were used for sampling. Four pitfall traps were set on each outside corner of each plot giving a total of 80 pitfall traps per field. Pitfall traps set up inside the OTCs were located on the 25 cm margin around the plot (see also in Experimental design). Pitfall traps consisted in a 330 ml plastic cup, about 12 cm deep and 8 cm in diameter at the top, filled with cars' cooling liquid at 20% ethylene glycol to which we added few drops of soap to break water tension. Pitfall traps were covered, using small iron sticks, with a plastic dish raised from the opening of the trap and letting it free of access. The dish protects the trap from eventual rain and avoids its overflow. Specimens collected were stored in ethanol (96%). For the analyses, the data from the four pitfalls of each plot were merged into one single sample giving a total of 10 replicates per treatment: 10 controls and 10 OTCs per field. Arthropod sampling was performed during the winter and summer 2020 using pitfall traps set for 14 days, with a single exception of Field B in summer for which traps were available 13 days. In this case, the 14^th^ day arthropod abundance was extrapolated from the other days. Species richness was not extrapolated.

All arthropods sampled were sorted and identified to species level, when possible, for the following target groups: Arachnida (Araneae, Opiliones, Pseudoscorpiones), Diplopoda, Chilopoda and Insecta (excluding Diptera, Hymenoptera - but including Formicidae - and Lepidoptera). Unidentified specimens were assigned to a morphospecies code. Initial sorting and identification were performed by the first author (SW) and students acting as parataxonomists (see list in Acknowledgements) and then by an expert taxonomist (PAVB). The nomenclature and colonisation status of the species follows the recent checklist of the Azorean arthropods (Borges et al. 2022). The analyses were conducted using the abundance of adult individuals identified to morpho(species) level. Despite the fact that, in previous studies on Azorean arthropods, juvenile spiders were commonly used (e.g. Cardoso et al. (2009)), in our study, the dominance of Erigoninae linyphiid spiders made this approach more difficult. All specimens are stored in Dalberto Teixeira Pombo (DTP) Collection in University of Azores (Terceira Island). Data are available in Wallon et al. (2023) (direct data access at: http://ipt.gbif.pt/ipt/resource?r=pasturclim_otc).

In intensive pastures of Terceira, exotic arthropods are usually predominant and the abundance of native and/or endemic species are reduced due to the high level of disturbance of the land (Cardoso et al. 2009, Rigal et al. 2017). Thus, as most of the species involved in our study are considered introduced for the Azores, no importance was given to the biogeographical origin of species in the analysis.

### Data analysis

We combined the catch data from the four traps present in each plot. In cases where a trap was damaged (not more than one per sampling event) and data were lost, theoretical abundances were estimated by extrapolating from the data from the other three traps. In addition, when traps were only operational for 13 days, we extended the sampling period to 14 days by extrapolating arthropod abundances.

In both cases, if a trap were damaged or the sampling period were shortened by one day, the species richness was considered to be the same. Only arthropod abundances were extrapolated.

Given the relatively low distances between experimental plots, we could not rule out the possibility of spatial autocorrelation amongst samples. As the precise coordinates of the experimental units were unknown, we established an artificial grid system at each site, using the measured distances between plots and the site coordinates (see above) serving as the reference point for the centre of the initial plot located in the top left corner of the grid. These calculated coordinates for the centre of each plot were then utilised to calculate the Moran's I values as a means of testing for spatial autocorrelation. Since our samples were, indeed, found to be spatially autocorrelated and they were also obtained through a repeated sampling protocol of the same sites, we incorporated both temporal and spatial autocorrelation into our model-building process and employed generalised linear mixed models (GLMMs) with multivariate normal random effects with Penalised Quasi-Likelihood, using the glmmPQL() function from the MASS package (Venables and Ripley 2002).

As response variables, we calculated the total activity-densities for each sampling event (i.e. the pooled number of arthropods from the four traps in summer or winter), as well as the first four Hill numbers (q = 0-3). Hill numbers (Legendre and Anderson 1999, Oksanen 2022) are diversity measures that summarise the number and relative abundance of different species in a community into a single value. The degree of importance given to the more or less dominant species increases with the q parameter. Hill number 0 represents the number of species in the community, whilst Hill numbers 1, 2 and 3 are equal to the exponential of the Shannon entropy index, the inverse Simpson index and the inverse Berger-Parker index, respectively.

We included treatment as a fixed variable, sampling season as a random variable and generated a correlation structure from the coordinates of the sampling plots to account for spatial autocorrelation. Count data were analysed using a Quasi-Poisson distribution, whilst for the Hill numbers, a Gaussian distribution (with a log link) was used. The same modelling approach was used for all captured arthropods and spiders and beetles separately.

Partial distance-based redundancy analysis (db-RDA, Legendre and Anderson (1999)) was used to investigate the effect of the treatment on arthropod community structure. Similarly to the GLMM approach, the sampling season and the exact location of the plots (as x and y coordinates) were considered as random effects and were partialled out using the “Condition” term in the capscale function from the vegan (version 2.6-4, Oksanen (2022)) package. Jaccard distances were used to compare communities and the significance of the model and the treatment effects were tested using a permutational ANOVA, with 999 iterations. Similarity percentage (SIMPER) analysis was used to identify the arthropod taxa that contributed the most to the observed difference between the treatments (Clarke 1993).

We also calculated beta diversity using the Jaccard Index to test the homogeneity in species composition between plots and seasons.

## Data resources

Data are available in Wallon et al. (2023) (direct data access at: http://ipt.gbif.pt/ipt/resource?r=pasturclim_otc).

## Results

### General patterns on species abundance

**Figure 3.**
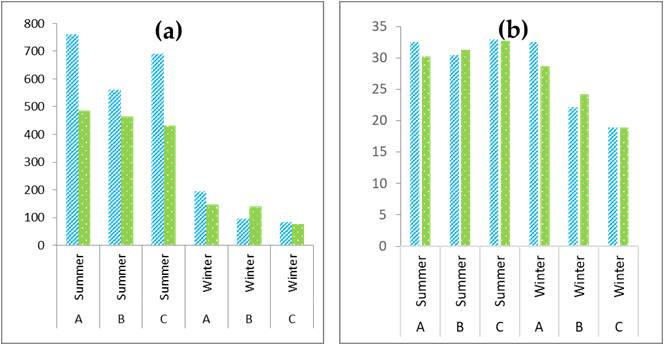
Mean abundance (a) and mean number of species (b) of arthropods collected in the two treatments (OTC in green dots, Control in blue stripes) for the three fields (A, B and C) during the summer and the winter.

Overall, we collected 41,351 specimens belonging to four classes, 15 orders, 60 families and 171 morphospecies. Of these, 34 taxa were only identified at order, family or genus level, resulting in 137 taxa with scientific species names associated (n = 38,918) (from now on “species”). Abundances were generally lower in winter than in summer, while no clear differences were present in terms of species richness (Fig. 3).

Introduced species (including those with indeterminate colonisation status, but still likely being exotic species (n = 7622)) represented 71% (n = 29664 specimens) of the total abundance and 75% (129 species) of the total richness; native non-endemic species represented 28% (n = 11608 specimens) of the total abundance and 19% (33 species) of the total richness; endemic species represented 0.2% (n = 79 specimens) of the total abundance and 1% (one species) of the total richness.

The two most diverse and abundant groups were spiders (Arachnida, Araneae) and beetles (Insecta, Coleoptera).

Overall, the omnivorous ground beetle *Pseudoophonusrufipes* (De Geer, 1774) (Coleoptera, Carabidae) dominated the samples and accounted for 17% of the total arthropod abundance. Besides the overall dominance, this species also dominated the summer samples, but the *Ocypusolens* (Müller, 1764) (Coleoptera, Staphylinidae) predatory rove beetle became dominant in the winter samples.

*Oedothoraxfuscus* (Blackwall, 1834) (Araneae, Linyphiidae) was the dominant spider, representing 5% of the overall arthropod abundance. It was also the most abundant spider species in summer samples, whilst the winter samples were dominated by *Erigonedentipalpis* (Wider, 1834) (Araneae, Linyphiidae).

Of the 20 most common species, four are considered native (*Leiobunumblackwalli*, *Tetramoriumcaespitum*, *Hypoponeraeduardi*, *Homalenotuscoriaceus*), one of undetermined origin (*Lithobius* sp.) and the other fifteen as introduced in the Azores.

Although not significantly, the structure of the most dominant species varied slightly with elevation and, therefore, with the type of field. In the low altitude field (field A), the ground-beetle *Notiophilusquadripunctatus* Dejean, 1826 (Coleoptera, Carabidae) dominated the winter samples (n = 464, 14%) and the European earwig *Forficulaauricularia* Linnaeus, 1758 (Dermaptera) the summer samples (n = 3177, 24%); at the intermediate altitude field (field B), the rove beetle *Ocypusolens* (Müller, 1764) (n = 579, 25%) dominated the winter samples and the ground beetle *Pseudoophonusrufipes* (De Geer, 1774) (n = 5822, 61%) the summer samples; and in the upper altitude field (field C), the rove beetle *Amischaanalis* (Gravenhorst, 1802) (Coleoptera, Staphylinidae) was the most abundant species during winter (n = 211, 14%), while the harvestman *Leiobunumblackwalli* (Arachnida, Opiliones) (n = 3882, 33%) was dominant in summer.

### Effects of OTC treatment on species composition and abundances patterns

**Figure 4.**
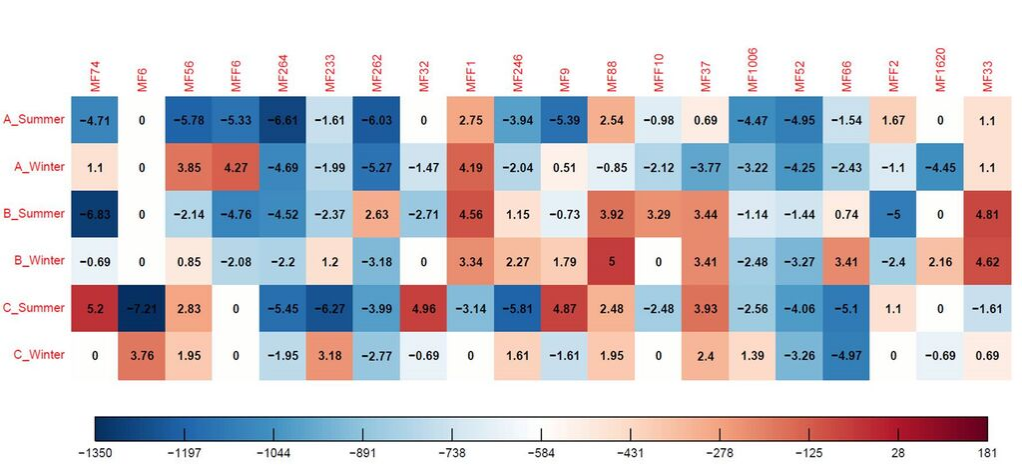
Heatmap showing the correlation between abundances and samples of the twenty most common species in all sites for different seasons. The colours and the corresponding values of the heatmap are logarithmic numbers of the relative species abundance. The coloured bar in the legend has been converted back to species abundance counts. The species abundance counts indicate the abundance differences between the control plots and the OTCs. Blue colours tend to indicate a lower abundance inside the OTCs, while reddish colours indicate higher abundances inside the OTCs. A, B and C correspond to the three pastures followed by the sampling season. On the heatmap, species appear as morphospecies (MF). From left to right, they correspond to the following: MF74 *Pseudoophonusrufipes* (Coleoptera); MF6 *Leiobunumblackwalli* (Opiliones); MF56 *Forficulaauricularia* (Dermaptera); MF F6 *Tetramoriumcaespitum* (Hymenoptera); MF 264 *Anotylusnitidifrons* (Coleoptera); MF 233 *Oedothoraxfuscus* (Araneae); MF 262 *Rugilusorbiculatus* (Coleoptera); MF 32 *Pterostichusvernalis* (Coleoptera); MF F11 *Solenopsis* sp. (Hymenoptera); MF 246 *Erigonedentipalpis* (Araneae); MF 9 *Ommatoiulusmoreleti* (Julida); MF 88 *Ocypusolens* (Coleoptera); MF F10 *Hypoponera* sp. (Hymenoptera); MF 37 *Polydesmuscoriaceus* (Polydesmida); MF 1006 *Lithobius* sp. (Lithobiomorpha); MF 52 *Cordaliaobscura* (Coleoptera); MF 66 *Amischaanalis* (Coleoptera); MF F2 *Hypoponeraeduardi* (Hymenoptera); MF 1620 *Notiophilusquadripunctatus* (Coleoptera); MF 33 *Homalenotuscoriaceus* (Opiliones).

According to the fitted GLMMs, the effect of OTCs treatment on arthropods abundance was significant (t = -4.88, p < 0.001), with a decrease in abundance in the OTC treatment compared to the control plots. The variance of the random intercepts was 0.62 (SD = 0.78) for the season and 43.90 (SD = 6.63) for the residual. However, the effect of OTCs treatment on arthropod richness (which also correspond to the Hill number, q = 0) was not significant (t = 0.34, p = 0.73). The variance of the random intercepts was 0.02 (SD = 0.15) for the season and 44.79 (SD = 6.69) for the residual. Fig. 4 visualises the abundance differences of the twenty most abundant species between sampling sites and seasons. Bluish colours indicate lower abundances inside the OTCs, while reddish colours indicate higher abundances inside the OTCs.

**Figure 5.**
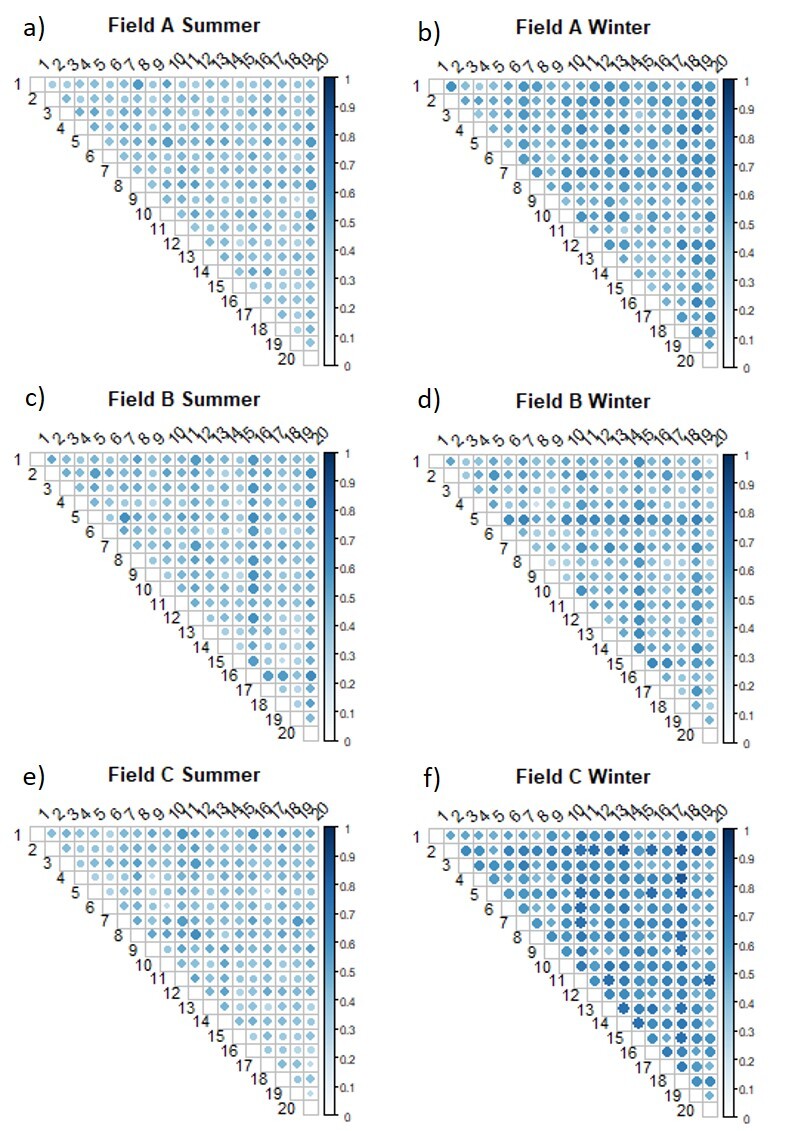
Heatmaps of the dissimilarities from the pairwise beta diversity analyses with using a Jaccard index a) Field A in summer, b) Field A in winter, c) Field B in summer, d) Field B in winter, e), Field C in summer, f) Field C in winter. Numbers 1 to 10 correspond to the 10 Control plots and 11 to 20 correspond to the 10 OTCs. Colour scale ranging from light to dark blue indicates increasing levels of dissimilarity. On the right side of the heatmap, the legend colour shows the dissimilarity values and the corresponding colours.

Dissimilarities between plots (Fig. 5) showed a seasonal variation, with greater dissimilarities (high β-diversity values) between winter plots than between summer ones.

**Figure 6.**
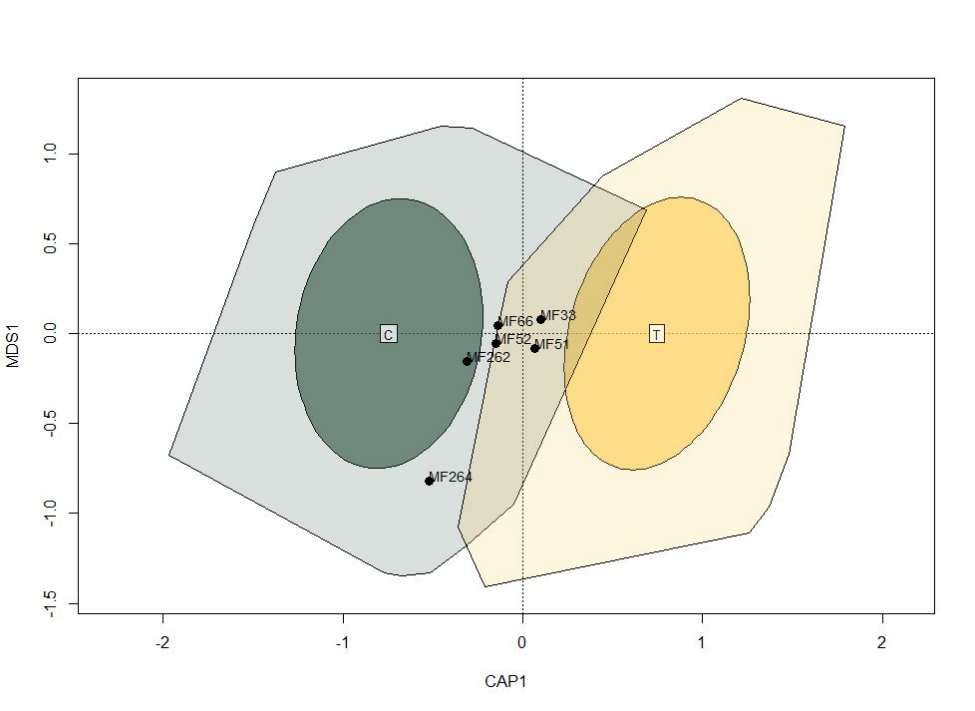
Partial distance-based redundancy analysis. Coloured hulls represent the ordination space in which all samples are included and ellipses represent areas in which 75% of the samples are included. Green colour (C) corresponds to control and yellow colour (T) corresponds to the OTC treatment. Species most responsible for differences between treatments are marked: MF33 *Homalenotuscoriaceus* (Simon, 1879); MF51 *Paranchusalbipes* (Fabricius, 1796); MF52 *Cordaliaobscura* (Gravenhorst, 1802); MF66 *Amischaanalis* (Gravenhorst, 1802); MF262 *Rugilusorbiculatus* (Paykull, 1789); MF264 *Anotylusnitidifrons* (Wollaston, 1871).

Those differences between control and OTCs were confirmed by the db-RDA (Fig. 6) that showed a significant difference in species composition between the arthropod communities sampled in OTCs versus those sampled in control plots (F = 3.2096, p < 0.001). The SIMPER analysis identified the harvestman, *Homalenotuscoriaceus* (Simon, 1879) (MF33) and the beetles *Paranchusalbipes* (Fabricius, 1796) (MF51), *Cordaliaobscura* (Gravenhorst, 1802) (MF52), *Amischaanalis* (Gravenhorst, 1802) (MF66), *Rugilusorbiculatus* (Paykull, 1789) (MF262) and *Anotylusnitidifrons* (Wollaston, 1871) (MF264) which contributed the most to the observed difference between the two treatments. Determining the most influential species in separating the treatments was based on the Similarity Percentage analysis developed by Clarke (Clarke 1993). Essentially, this method estimates the mean contribution of a species in separating two groups based on (dis)similarity changes in the cluster analysis when the species is removed from the samples. In our case, since we used abundance-based Bray-Curtis distances, the presence or absence of a species either in the treatment or the control could have been only influential if the difference in the abundance had been large as well. Since, in most cases, rare species showed presence-absence differences, the method highlighted other species, those which were present in both treatments, but showed the largest abundance differences between treatments. It is clear that these species are mostly in the overlapping area in the hull, but outside of the ellipses where 75% of the points are.

When GLMMs were applied to a subset of the data containing beetles only, the results indicated a significant negative effect of OTC treatment on both the beetles’ abundance (estimate = -0.36, SE = 0.07, t = -5.39, p < 0.001) and species richness (estimate = -0.102, SE = 0.039, t = -2.619, p = 0.01). The variance components showed that the effect of the season accounted for a small, but significant proportion of the variance of beetle abundance (0.35, SE = 0.12), while the variance components of the beetle species richness GLMM highlighted a significant seasonal variation in beetle species richness (variance < 0.001, SD < 0.001). This indicates that there was a significant seasonal variability both in beetles’ richness and abundance.

For spiders, the abundance was significantly lower in OTC treatments (estimate = -0.23, SE = 0.11, t = -2.10, p < 0.001). On the other hand, OTCs had a significantly positive effect on spider richness (estimate = 0.11, SE = 0.04, t = 3.03, p < 0.001).

### Effects of OTC treatment on species relative abundance patterns

The analysis of the Hill number series (Table 1) found no significant difference in the exponential of the Shannon entropy (q = 1) between the control and OTC plots either for all arthropods or for beetles. This value, however, showed a highly significant difference for spiders (p = 0.002). Diversity curves (Gotelli and Chao 2013) (Suppl. material 1, Suppl. material 2, Suppl. material 3) highlighted a trend for a higher evenness of spider communities inside OTCs.

For q = 2, which represents the inverse of the Simpson index, there was a significant difference between the control and OTC plots for spiders (p = 0.021). However, there was no significant difference between the control and OTC plots for beetles and when considering all arthropods.

For q = 3, which represents the inverse of the Berger-Parker index, there was no significant difference between the control and OTC plots for all arthropods, beetles or spiders.

## Discussion

Disentangling the impact of temperature increase on biodiversity patterns in agroecosystems constitutes a fundamental research challenge. During this short-term experiment, we simulated an increase in temperature using OTCs and tested the impacts on arthropods occurring on intensively-grazed Azorean pastures.

Most of the species we caught were introduced, commonly distributed in agroecosystems. Indeed, since highly-disturbed habitats, such as Azorean intensive pastures, mainly select for species with high dispersal capacities which can rapidly colonise ecosystems and respond to disturbances, less flexible indigenous species often have a competitive disadvantage and the presence of one or few dominant exotic species is common (Cardoso et al. 2009, Meijer et al. 2011, Rigal et al. 2017).

The OTCs had a significant negative effect on both the abundance and diversity of arthropods, suggesting that increased-temperature environments are less favourable for most arthropods in these pastures. Additionally, the study found that treatment effect was not uniform for all arthropods, but differed between taxa, with beetles and spiders showing different trends under the OTC treatment. Consequently, some taxa may be more vulnerable to global warming than others, which, in turn may influence the ecosystem services they deliver or the disservices they cause. Indeed, Thakur et al. (2017) found that warming can increase predation effects and reduce the co-existence of prey, while Moss and Evans (2022) demonstrate how it can negatively impact arable farming systems, potentially harming wildflowers and insects that rely on them. In addition, Skendžić et al. (2021) suggest that warming can also lead to an increase in pest abundance and promote the spread of invasive species.

### Effects of OTCs on species composition on arthropods and abundances patterns

Our results indicate that the composition of the entire arthropods community was impacted by the OTC treatment. Although the richness of the overall community was not affected, in the case of particular taxa, we noticed signs of changes that should be confirmed over much needed long-term experiments.

Indeed, some common agroecosystems species, such as *Paranchusalbipes*, *Cordaliaobscura*, *Amischaanalis*, *Rugilusorbiculatus*, *Anotylusnitidifrons* and *Homalenotuscoriaceus*, contributed disproportionately more to the observed differences than other species.

Beetles’ richness (mostly composed of carabids and rove beetles) was lower inside the OTCs and they were found to be more diverse in control plots. Although Thiele (1977) identified temperature and humidity amongst the most influential abiotic factors for carabid populations, a direct avoidance of warmer sites was not reported. Several carabid species, however, have been reported to decline with warming (Müller-Kroehling et al. 2014) and, since most ground beetle species inhabiting agricultural fields are mostly eurytopic and present a considerable dispersal ability (Holland 2002), they likely can avoid unsuitably hot areas. Rove beetles, similarly to carabids, have good dispersal abilities (Halder 2013) and the capacity to avoid unsuitable areas. They, on the other hand, are often thermophilous and tend to develop faster in higher temperatures than in colder conditions (Irmler et al. 2018).

In contrast to beetles, spiders’ richness (which belong mostly to the linyphiids) was higher inside the OTCs. This could be explained by a higher plant biomass and vegetation structural complexity inside the OTCs in our experiment (Melo et al. 2022) which favours a greater diversity of predators and, therefore, of spiders. Indeed, Borges and Brown (2001) and Borges and Brown (2004) found that, in Azorean pastures, linyphiids, that build their web close to the ground, tend to occur more in dense and structurally diverse grass that provide more structure for shelters and web attachments. In our experiment, the main linyphiid species sampled were *Oedothoraxfuscus* (Blackwall, 1834), *Erigonedentipalpis* (Wider, 1834), *Erigoneatra* Blackwall, 1833, *Erigoneautumnalis* Emerton, 1882 and *Tenuiphantestenuis* (Blackwall, 1852) which are agrobiont species very common in cultivated areas, agroecosystems and disturbed areas (Downie et al. 2000, Thorbek et al. 2003, Blandenier 2009, Harper 2020). On the other hand, no effect of the vegetation was observed in other taxa in our experiment. This might be due to the fact that pitfalls traps catch ground-dwelling taxa and species that live in higher strata of the vegetation were not the target of our experiment. If sampling had been conducted in the upper layers of vegetation, with greater plant biomass, higher arthropod abundances could also have been expected (Prather and Kaspari 2019, Prather et al. 2020).

The overall abundance, as well as that of beetles and spiders, were negatively impacted by the OTCs. Yet, the responses of different arthropod groups cannot be easily generalised. Indeed, in our study, the diversity of spider increased inside the OTCs, whilst that of beetles decreased. Thus, in Azorean pastures, spiders and beetles appear to respond differently to an increase in temperature.

Our study agrees with Kwon et al. (2015) who showed that more beetle species will decrease rather than increase as the climate warms. Although, in their paper, Kwon et al. (2015) report a decrease of the rove beetle species *Ocypuscoreanus* with warming climate, this contradicts our results where *O.olens*, a species from the same genus, was found in higher abundances in treated plots. Therefore, the two species seemingly react differently to abiotic changes.

Besides taxa responding differently to the heating treatment, the community composition also showed seasonal differences: we observed higher beta diversity values in winter when all species were considered in the community. This pattern might be explained by the fact that most arthropods in the Azores have a reduced abundance during the winter and tend to peak during the summer (Borges 1995, Borges et al. 2017) and the fewer individuals in samples allow a greater influence of stochastic processes in community assembly, which, in turn, might create more disparities between plots.

### Effects of OTCs on species’ relative abundances patterns

Species diversity is influenced not only by the number of species present in a community and their overall abundances, but also how individuals are distributed amongst those species. Although other studies, in which similar taxa were monitored as our experiment, found that certain species of ground beetles, spiders and Hemiptera became superabundant and the evenness declined with the rise in temperature (Buchholz et al. 2012, Høye et al. 2021, Skendžić et al. 2021), our results did not show this pattern. In our study, the Berger-Parker indexes did not reveal a clear dominance pattern and our results agree with those of Tsafack et al. (2019), who observed a negative effect of temperature on dominant arthropods in northern Chinese grasslands. Furthermore, OTCs had a significantly positive impact on spider evenness, suggesting that the treatment is promoting the survival and persistence of a wide range of spider species, rather than just a few dominant species. This may be caused by either the changes in microclimatic conditions or vegetation structure caused by the OTC (e.g. higher plant biomass, Borges and Brown (2001), Borges and Brown (2004)) which could provide more favourable conditions for some species.

Yet, this reduction in dominance and the increase in evenness, was statistically significant for spiders only. Indeed, even though a decrease in beetle abundance was observed with the GLMMs, Hill number analysis surprisingly did not reveal statistical differences and neither it did when all arthropods were considered. Other taxa not analysed here separately or differences between species’ ecology could also play a role in masking the trends.

Indeed, as our results showed, responses to increased temperature can be variable from one taxonomic group to the other and from one species to the other. Undoubtedly, species balance between their responses to higher temperature in the altered environment, their optimum thermal conditions and resource (e.g. food or habitat) availability and this balance highly depends on species’ traits. Paler et al. (2021) found variations in beetle diversity with OTCs treatment when looking at their feeding behaviour, body size and colour. Moreover, as arthropods are ectotherms, changes in their physiology and metabolism under heat stress can depend on their developmental stages or locomotion capacities (Garcia and Clusella-Trullas 2019). Diel activity may also have an impact on the arthropod responses to increased temperature and nocturnal species can be less prone to daylight heat effects than diurnal ones because they remain hidden from the direct solar exposition and extreme temperatures (Thiele 1977). Altogether, considering species’ ecology (i.e. traits) is key for a mechanistic understanding of the processes driving community-wise adaptations to warming. Thus, to disentangle the impacts of OTCs on the arthropod community, future investigation focusing on functional traits are necessary.

This can be particularly important with species of great economic importance (e.g. pests or natural enemies). For example, in the context of increased temperature, Hannigan et al. (2022) observed that the pest *Sitonagressorius* (Fabricius, 1792) has its activity (dispersal and feeding rate) increased. With climate change, this represents a risk of pest spreading in agroecosystems (Tubiello et al. 2008). A similar species in our study, *Sitonadiscoideus* Gyllenhal, 1834, that is also considered a plant pest in different parts of the world (Kean and Barlow 2002, Goldson et al. 2009), however, did not show a positively correlating abundance trend with the increasing temperature.

Additionally, since significant changes in species composition over a prolonged period can increase the probability of altered ecosystem functions (Cardinale et al. 2012), to monitor these changes and to clarify effects of temperature on arthropod communities on a larger temporal scale, longer-term experiments are increasingly needed.

Although our experiment was successful to predict some impacts of climate change on Azorean arthropods communities, some limitations may apply. The OTCs seems to have an indirect effect on the arthropod communities: they had an effect on the vegetation structure, particularly by increasing plant biomass (unpublished data), which may affect our results. These effects, however, need to be confirmed with further investigations. Moreover, Nash et al. (2013) warn that OTCs can reach extreme temperatures during the day that do not reflect the average increased temperature commonly used in climate change research. The two climate scenarios on which we based our experimental set-up (e.g. RCP 4.5 and RCP 8.5, PRAC 2017) are the intermediate and worst-case scenarios. Although these are widely used in climate change research, Pielke and Ritchie (2021) highlighted that overly emphasising the worst-case scenarios may potentially overestimate the impacts; thus, their assessment requires careful consideration and critical evaluation.

## Conclusions

Our results suggest that the simulated warming had a significant impact on arthropod communities in the study area, by affecting their species richness, evenness and dominance structure. However, the impact varies depending on the arthropod group or even from species to species.

Although our study provides some important insights into the impact of increased temperature on Azorean pasture arthropods, more research is needed to allow a deeper insight. For instance, the comparison of this result with a simulated increase in temperature along an altitudinal gradient, as well as a long-term study, could help to untangle the impacts of increased temperatures in varying environments and on species with different cold adaptations. In addition, more studies on the ecology and functional traits of selected key species in Azorean pastures could help to predict general arthropod population trends for the future.

## Supplementary Material

AAB2C5A2-0FC8-5336-983D-F6FE3DB016B8 10.3897/BDJ.11.e107385.suppl1 8416365 Supplementary material 1 Diversity curves using Hill numbers for arthropod assemblages in the Fields A, B and C for both seasons, winter and summer Data type image Brief description Diversity curves using Hill numbers for arthropod assemblages in the Fields A, B and C for both seasons, winter and summer. a) FAW: Field A Winter; b) FAS: Field A Summer; c) FBW: Field B Winter; d) FBS: Field B Summer; e) FCW: Field C Winter; f) FCS: Field C Summer. q orders are shown on the x axis, q0 = species richness; q1 = exponential Shannon diversity index; q2 = inverse Simpson diversity; q3 = inverse Berger-Parker diversity and their corresponding value on the y axis. Blue curves correspond to the control plots and green dotted curves to the OTCs plots. File: oo_856635.png https://binary.pensoft.net/file/856635 Sophie Wallon, Noelline Tsafack, Gabor Pozsgai, Catarina D. Melo, Paulo A. V. Borges and Rui B. Elias

66C575CA-46EC-5392-8EAC-761B28137E40 10.3897/BDJ.11.e107385.suppl2 8416369 Supplementary material 2 Diversity curves using Hill numbers for beetle assemblages in the Field A, B and C for both seasons, winter and summer Data type image Brief description Diversity curves using Hill numbers for beetle assemblages in the Fields A, B and C for both seasons, winter and summer. a) FAW: Field A Winter; b) FAS: Field A Summer; c) FBW: Field B Winter; d) FBS: Field B Summer; e) FCW: Field C Winter; f) FCS: Field C Summer. q orders are shown on the x axis, q0 = species richness; q1 = exponential Shannon diversity index; q2 = inverse Simpson diversity; q3 = inverse Berger-Parker diversity and their corresponding value on the y axis. Blue curves correspond to the control plots and green dotted curves to the OTCs plots. File: oo_856636.png https://binary.pensoft.net/file/856636 Sophie Wallon, Noelline Tsafack, Gabor Pozsgai, Catarina D. Melo, Paulo A. V. Borges and Rui B. Elias

B3AC08D6-B7BA-5F71-8846-98C91D61A996 10.3897/BDJ.11.e107385.suppl3 Supplementary material 3 Diversity curves using Hill numbers for spider assemblages in the Fields A, B and C for both seasons, winter and summer Data type image Brief description Diversity curves using Hill numbers for spider assemblages in the Fields A, B and C for both seasons, winter and summer. a) FAW: Field A Winter; b) FAS: Field A Summer; c) FBW: Field B Winter; d) FBS: Field B Summer; e) FCW: Field C Winter; f) FCS: Field C Summer. q orders are shown on the x axis, q0 = species richness; q1 = exponential Shannon diversity index; q2 = inverse Simpson diversity; q3 = inverse Berger-Parker diversity and their corresponding value on the y axis. Blue curves correspond to the control plots and green dotted curves to the OTCs plots. File: oo_856637.png https://binary.pensoft.net/file/856637 Sophie Wallon, Noelline Tsafack, Gabor Pozsgai, Catarina D. Melo, Paulo A. V. Borges and Rui B. Elias

## Figures and Tables

**Table 1.** Results of the t-value extracted from the glmmPQL in order to compare Hill Number values considering all arthropods, beetles and spiders. Hill number q1 represents the exponential of the Shannon entropy index, whilst q2 and q3 correspond to the inverse Simpson index and the inverse Berger-Parker index, respectively. t-values indicate the trends (negative or positive) inside the OTCs.

	**All arthropods**		**Beetles**		**Spiders**	
	t-value	p-value	t-value	p-value	t-value	p-value
q1	-0.069	0.945	-1.893	0.061	3.226	0.002
q2	-0.345	0.731	-1.818	0.072	2.33	0.021
q3	0.096	0.923	-1.801	0.074	1.862	0.065
