# Supplementary figures and images for: Effects of a short-term temperature increase on arthropod communities associated with pastures
Source: Biodivers Data J. 2023 Oct 5;11:e107385. doi: 10.3897/BDJ.11.e107385 (PMC10570815; doi:10.3897/BDJ.11.e107385)

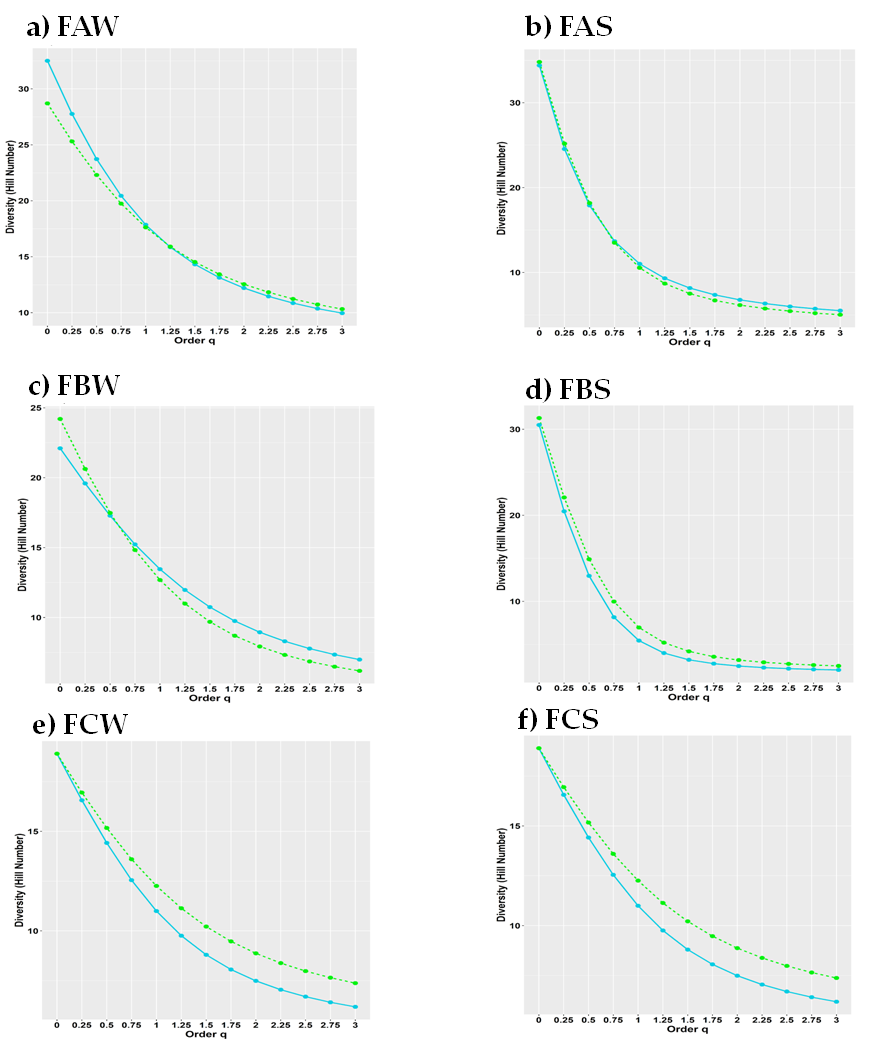

Supplement: Supplementary material 1 — Diversity curves using Hill numbers for arthropod assemblages in the Fields A, B and C for both seasons, winter and summer [file bdj-11-e107385-s001.png]

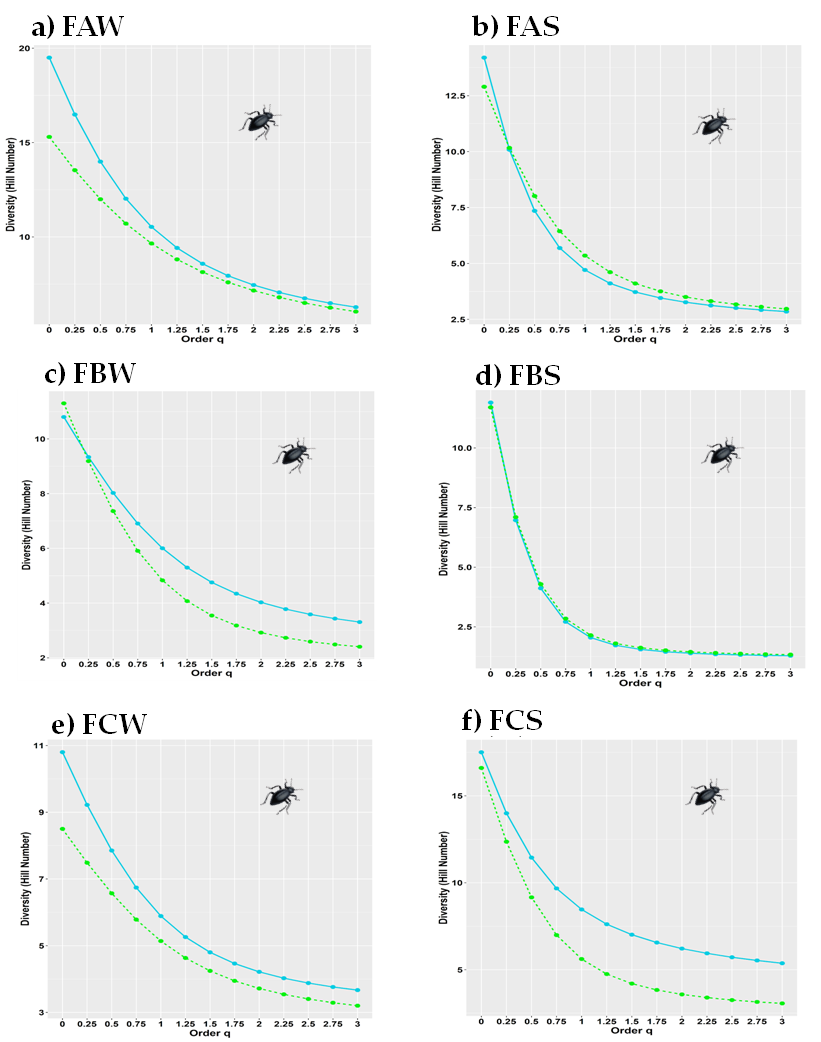

Supplement: Supplementary material 2 — Diversity curves using Hill numbers for beetle assemblages in the Field A, B and C for both seasons, winter and summer [file bdj-11-e107385-s002.png]

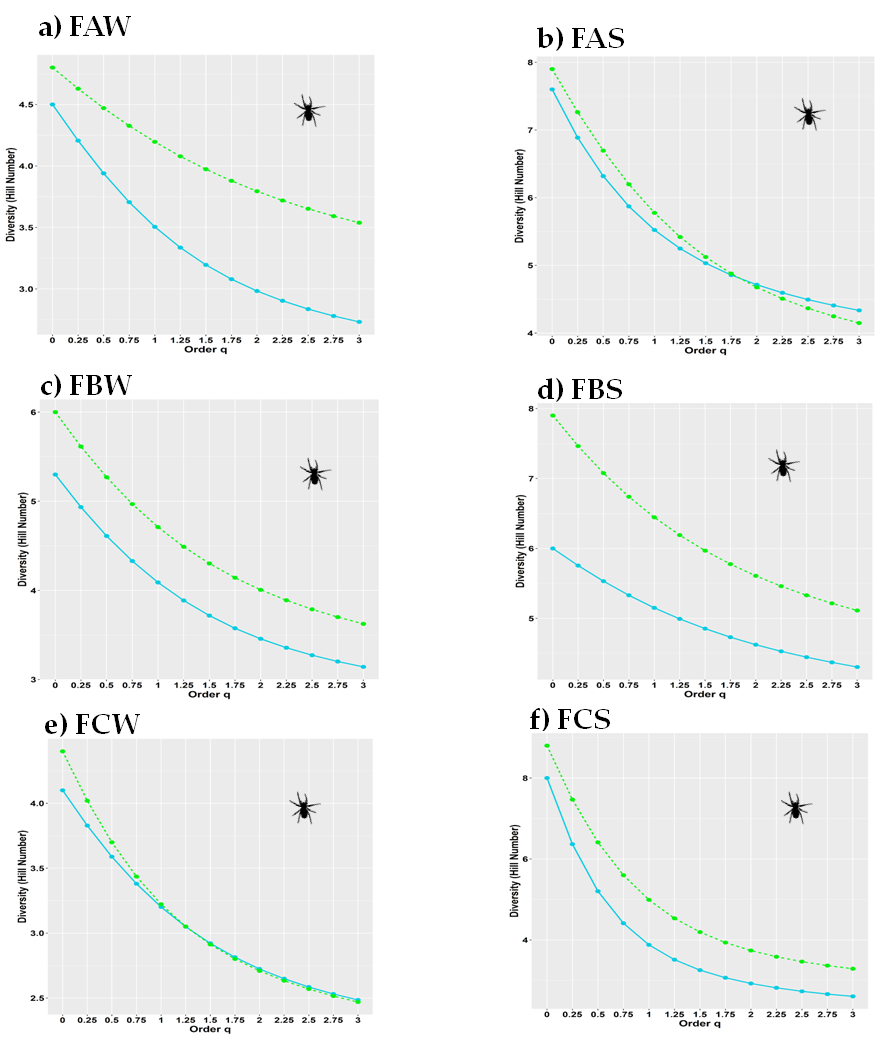

Supplement: Supplementary material 3 — Diversity curves using Hill numbers for spider assemblages in the Fields A, B and C for both seasons, winter and summer [file bdj-11-e107385-s003.png]
